# Creator Type, Content Format, and Engagement Among Highly Liked Cancer‐Related TikTok Search Results: A Cross‐Sectional Content Analysis

**DOI:** 10.1002/cnr2.70734

**Published:** 2026-10-30

**Authors:** Eunice Y. Ju, Esther D. Ju, Russell G. Witt

**Affiliations:** ^1^ University of Virginia School of Medicine Charlottesville Virginia USA; ^2^ University of Maryland School of Dentistry Baltimore Maryland USA; ^3^ Department of Surgery University of Virginia Charlottesville Virginia USA

**Keywords:** cancer information, cross‐sectional content analysis, health communication, social media, TikTok

## Abstract

**Background:**

Engagement differences by creator type among highly liked cancer‐related TikTok videos remain unclear.

**Methods:**

We analyzed 250 videos from five cancer searches, selecting the first 50 results per search filtered to the preceding six months and sorted by likes. Health professionals or trainees were classified as medical. The primary analysis modeled log‐transformed views by creator type, adjusting for followers, video age, content type, and cancer search.

**Results:**

Adjusted views showed no significant difference (ratio, 0.90; 95% CI, 0.44–1.85; *p* = 0.773). Medical creators had a higher adjusted share rate (ratio, 2.01; 95% CI, 1.24–3.24; Holm‐adjusted *p* = 0.026). Median views per follower were lower for medical creators (2.55 vs. 16.40; ratio, 0.16; 95% CI, 0.06–0.45; Holm‐adjusted *p* = 0.003).

**Conclusions:**

Despite no clear difference in absolute views, lower views per follower raise the possibility that medical creators have more limited reach beyond established audiences than nonmedical creators, while greater sharing may broaden distribution.

## 1 Introduction

TikTok hosts cancer‐related content that accumulates billions of views and has become an important source of health information for young adults [1]. Cancer misinformation on social media platforms such as TikTok is associated with harmful health beliefs and treatment decisions [2]. Medical professionals have established TikTok presences to provide evidence‐based cancer information and correct harmful misinformation. Although accuracy remains important, the potential public health contribution of clinicians also depends on how their content performs relative to the size of their existing audience and how viewers engage with it. To our knowledge, how creator identity and content format relate to view counts and engagement has not been characterized for cancer‐specific content on TikTok.

TikTok recommendations are shaped by user engagement including follows, watch time, likes, and comments [3]. We focused on highly liked videos because they represent cancer‐related content that had already successfully attracted substantial attention. Within this selected set, examining views, comments, shares, and saves separately may show whether audiences engage differently with content from medical and nonmedical creators or across content formats.

We compared absolute views between medical and nonmedical creators, adjusting for measured covariates, and examined views per follower, engagement measures, and content patterns as secondary or descriptive outcomes.

## 2 Methods

We identified TikTok videos using keyword searches on April 28–29, 2026, between approximately 6:00 to 10:00 PM. Searches were conducted in the TikTok mobile application version 44.4.0 on a single Apple iPhone 16 Pro while logged out of a TikTok account. The device language was set to English, region settings to United States, and all searches were performed in a single city in Maryland. Search terms were entered in the following order: “skin cancer,” “breast cancer,” “colon cancer,” “lung cancer,” and “prostate cancer.” The terms were selected as plain‐language, site‐based labels. Search history and app cache were not cleared between searches. Within each search, TikTok's in‐app filters were applied to restrict results to videos posted within the preceding 6 months and to sort results by like count. For each cancer type, the first 50 visible videos returned after applying these filters were included. Individual displayed ranks of each video and creator identities were not retained and no duplicate videos were present. Because TikTok does not disclose how it ranks filtered search results, we considered this a fixed‐size, stratified convenience sample of highly liked cancer videos rather than a definitive list of the platform's 50 most‐liked videos.

Creators were classified as medical only when their profiles self‐disclosed them as a health‐professional or trainee. Credentials were not externally verified. The 62 videos in this group included 50 from physicians, nurse practitioners, or nurses and 12 from one pharmacist, registered dietitian, ultrasound technician, neurophysiologist, naturopathic/chiropractic professional, public‐health PhD, neuroscience PhD student, four medical students, and one BSN student. The remaining 188 videos were classified as nonmedical creator content. A second reviewer independently classified all 250 videos using the same prespecified criteria. Initial agreement was 98.8% with high interrater reliability (Cohen *κ* = 0.97; 95% CI, 0.93–1.00). The three disagreements were resolved by consensus, with the original classifications retained. A four‐category sensitivity analysis separated physicians/nurse practitioners/nurses (*n* = 50), other health professionals/trainees (*n* = 12), patients/survivors without a recorded professional role (*n* = 122), and other creators (*n* = 66). A recorded professional role took precedence over patient/survivor status.

Videos were categorized into 9 predefined content types (fundraising, humor, lifestyle/dietary, personal experience/story, basic science research, etiology, prevention, treatment, and symptom‐focused) developed through preliminary review of cancer‐related TikTok content prior to formal data collection. Mixed videos were assigned to the dominant focus. For example, personal illness narratives were coded as experience, mechanistic studies as research, and screening or risk‐reduction guidance as prevention. To assess reliability of content type classification, a simple random sample of *n* = 50 was independently coded by a second reviewer; inter‐rater agreement was almost perfect (Cohen's *κ* = 0.91; 95% CI, 0.83–1.00). Content coders were aware of creator status, and engagement metrics were visible during coding.

Absolute views were the primary outcome. Log‐transformed views were modeled by creator category, adjusting for log‐transformed follower count, nonlinear video age, content type, and cancer search. Exploratory comparisons in Table 1, Panel A used two‐sided Mann–Whitney U tests with Holm correction across 12 comparisons. The six engagement outcomes in Table 2 were secondary, modeled with the same covariates, with Holm adjustment across outcomes. Views‐per‐follower comparisons were exploratory, and content‐type findings were descriptive; skewed metrics were summarized using medians and IQRs. Analyses used R version 4.5.2.

**TABLE 1:** Descriptive characteristics and views‐per‐follower analyses by creator category.

Characteristic	Health professional/trainee (*n* = 62)	Other creators (*n* = 188)	Holm‐adjusted *p*
*Panel A. Binary creator categories*			
Follower count	97 100 (10024‐541 800)	10 150 (2259‐93 800)	< 0.001
Video age, *d*	50 (25–91)	41 (17–86)	0.671
Video length, s	62 (31–116)	63 (23–150)	1.000
Posting frequency, past month	24 (14–33)	25 (8–42)	1.000
Views	330 300 (68675‐1 350 000)	280 450 (42900‐1 200 000)	1.000
Views per follower	2.55 (0.72–21.17)	16.40 (1.87–112.95)	0.003
Overall engagement rate, %	6.14 (2.48–11.84)	7.70 (3.54–13.28)	0.671
Like rate, %	4.85 (1.88–8.16)	6.52 (2.73–11.09)	0.302
Comment rate, %	0.08 (0.03–0.20)	0.12 (0.03–0.32)	0.137
Save rate, %	0.40 (0.20–1.00)	0.32 (0.16–0.61)	0.277
Share rate, %	0.27 (0.11–1.15)	0.23 (0.10–0.56)	0.459

Creator category	n	Views per follower, median (IQR)	Holm‐adjusted *p*
*Panel B. Four‐category views‐per‐follower sensitivity analysis*			
Individual clinicians	50	4.07 (0.72–25.96)	0.003
Other health professionals/trainees	12	1.45 (0.87–4.18)	0.013
Patients/survivors	122	20.69 (2.26–114.18)	Reference
Other creators	66	5.51 (0.61–101.43)	0.420

*Note:* Data are median (IQR) unless otherwise indicated. Panel A *p*‐values are from two‐sided Mann–Whitney *U* tests with Holm correction across 11 comparisons. Panel B *p*‐values compare each category with patients/survivors, with Holm correction across all six pairwise comparisons. Overall engagement rate = (likes + comments + saves + shares)/views × 100. Component rates are per 100 views.

Abbreviation: IQR, interquartile range.

**TABLE 2:** Adjusted primary and secondary models by creator category.

Outcome	Role	Adjusted ratio (95% CI)	*p*	Holm‐adjusted *p*
*Panel A. Binary creator‐category models*				
Absolute views	Primary	0.90 (0.44–1.85)	0.773	—
Overall engagement rate	Secondary	1.25 (0.84–1.85)	0.270	0.811
Like rate	Secondary	1.17 (0.80–1.71)	0.424	0.849
Comment rate	Secondary	1.27 (0.67–2.39)	0.468	0.849
Save rate	Secondary	1.50 (1.08–2.07)	0.015	0.073
Share rate	Secondary	2.01 (1.24–3.24)	0.004	0.026
*Panel B. Four‐category adjusted absolute‐view sensitivity model*				
Individual clinicians vs. other creators	Sensitivity	0.75 (0.34–1.69)	0.490	0.979
Other health professionals/trainees vs. other creators	Sensitivity	0.60 (0.07–4.95)	0.639	0.979
Patients/survivors vs. other creators	Sensitivity	0.65 (0.30–1.37)	0.252	0.757

*Note:* Ratios in panel A compare medical with other creators; panel B uses other creators as the reference. Models adjusted for log follower count, nonlinear video age, content type, and cancer search and used HC3 robust 95% CIs. The primary absolute‐view *p*‐value was not adjusted for multiple comparisons. Holm adjustment was applied across the 6 secondary outcomes in panel A and the 3 contrasts in panel B. A 0.5 correction was applied to all counts used in the engagement transformations.

Abbreviation: CI, confidence interval.

This study analyzed publicly accessible TikTok content without interaction with users. Public posts may contain identifiable health disclosures. This study was approved by the institutional review board.

## 3 Results

Among 250 high‐performing cancer‐related videos, 62 (24.8%) were produced by medical creators and 188 (75.2%) by nonmedical creators. Median absolute views were 330 300 (IQR, 68675–1 350 000) for medical creators and 280 450 (IQR, 42900–1 200 000) for nonmedical creators. In the primary multivariable model, adjusted absolute views showed no clear difference by creator type (ratio, 0.90; 95% CI, 0.44–1.85; *p* = 0.773). Median follower counts were 97 100 (IQR, 10024–541 800) and 10 150 (IQR, 2259–93 800), respectively. Views per follower were markedly lower for medical creators. The median was 2.55 (IQR, 0.72–21.17) for medical creators and 16.40 (IQR, 1.87–112.95) for nonmedical creators (Holm‐adjusted *p* = 0.003). The ratio of medians was 0.16 (95% CI, 0.06–0.45). Posting frequency (24 vs. 25 posts in the preceding month) and video length (62 vs. 63 s) were comparable between groups (Table 1).

In the four‐category sensitivity analysis, median views per follower were 4.07 for physicians/nurse practitioners/nurses, 1.45 for other health professionals/trainees, 20.69 for patients/survivors, and 5.51 for other creators (Kruskal‐Wallis *p* < 0.001). After Holm adjustment, patients/survivors differed from physicians/nurse practitioners/nurses (*p* = 0.003) and other health professionals/trainees (*p* = 0.013). No other pairwise comparison was significant (Table 1).

Adjusted overall engagement rate showed no clear difference by creator type (ratio, 1.25; 95% CI, 0.84–1.85; adjusted *p* = 0.811). Notably, medical creators had a higher adjusted share rate (ratio, 2.01; 95% CI, 1.24–3.24; adjusted *p* = 0.026); none of the other secondary component measurements remained significant after Holm adjustment (Table 2).

Personal experience/story was the most common content category (91 [36.4%]), followed by symptom‐focused (34 [13.6%]) and treatment content (33 [13.2%]). Fundraising videos had the highest median overall engagement rate (25.12%; IQR, 24.93%–26.27%; *n* = 5), followed by humor videos (16.65%; IQR, 9.65%–20.46%; *n* = 16). Because the fundraising category included only five videos, we treated these findings as descriptive and did not formally compare this category with the others (Table 3).

**TABLE 3:** Descriptive outcomes by content category.

Content category	Videos, *n* (%)	Professional/trainee, *n* (%)	Views, median (IQR)	Overall engagement rate, median (IQR), %
Fundraising	5 (2.0)	0 (0.0)	1 600 000 (208800‐10 900 000)	25.12 (24.93–26.27)
Humor	16 (6.4)	2 (12.5)	420 850 (65625‐876 050)	16.65 (9.65–20.46)
Lifestyle/dietary	20 (8.0)	5 (25.0)	278 350 (39550‐1 049 850)	12.00 (8.13–17.36)
Personal experience/story	91 (36.4)	8 (8.8)	241 700 (14100‐944 250)	8.21 (4.49–13.56)
Basic science research	9 (3.6)	6 (66.7)	134 100 (1845‐152 500)	7.25 (3.41–11.43)
Etiology	16 (6.4)	8 (50.0)	559 750 (293825‐2 950 000)	5.99 (3.00–7.45)
Prevention	26 (10.4)	15 (57.7)	334 300 (99300‐1 058 950)	6.11 (2.65–7.57)
Treatment	33 (13.2)	7 (21.2)	210 000 (70000‐1 200 000)	4.22 (2.36–8.19)
Symptom‐focused	34 (13.6)	11 (32.4)	464 100 (121675‐1 700 000)	3.79 (1.92–7.53)
Overall	250 (100)	62 (24.8)	—	—

*Note:* Content‐category values are descriptive; no omnibus or pairwise inference was performed. Estimates for fundraising (*n* = 5) and basic science research (*n* = 9) are imprecise.

Abbreviation: IQR, interquartile range.

## 4 Discussion

In this selected sample of highly liked cancer TikTok videos, the primary analysis found no clear difference in adjusted absolute views between medical and nonmedical creators, although the confidence interval was wide. Views per follower were substantially lower for medical creators, and medical creators had a higher adjusted share rate. Overall engagement did not clearly differ. Together, these results suggest that medical and nonmedical creators may achieve similar total visibility through different patterns of audience exposure and redistribution.

Views per follower was the most pronounced creator‐group difference. Medical creators had nearly 10 times the median follower count, although follower counts were recorded at data collection rather than when each video was posted. The lower views per follower among medical creators raises the question of whether their videos circulate less widely beyond existing follower networks. Although TikTok's recommendation system makes this a plausible hypothesis, our data cannot distinguish it from differences in follower counts, follower activity, or changes in audience size after posting [4]. The higher adjusted share rate among medical creators may represent a separate dissemination pathway, perhaps reflecting content that viewers considered useful or worth passing along. However, sharing motivations were not measured, and we could not determine whether shares generated additional exposure.

Personal experience/storytelling was the most common content category, illustrating the prominence of experiential communication within highly liked cancer‐related search results. Fundraising videos had the highest median overall engagement rate, followed by humor videos. These findings are descriptive, and the fundraising estimate was based on only five videos. Although these patterns characterize engagement with different types of cancer content, they do not explain how viewers interpreted the information.

The elaboration likelihood model (ELM) offers a framework for exploring this distinction by considering how message content and source credibility influence persuasion [5, 6, 7]. Applied to cancer‐related TikTok content, professional credentials could serve as credibility cues, while explanations of cancer prevention or treatment could provide arguments for viewers to evaluate. Either medical or nonmedical creators may present substantive arguments, and neither creator identity nor content format alone determines how viewers process a message. However, our cross‐sectional analysis captured video characteristics and aggregate engagement rather than viewers' responses to the information. We did not assess argument quality, viewer motivation or knowledge, cognitive processing, or attitude change, and engagement counts cannot establish elaboration. ELM therefore provides a framework for developing hypotheses about this content, but the available data could not test its proposed mechanisms.

The findings generate hypotheses for prospective communication research. Clinicians and public health organizations could test whether combining subject‐matter expertise with platform‐specific communication strategies such as concise openings, relatable examples, storytelling, visual explanations, audience‐oriented language, and clear calls to action improves attention without compromising accuracy. Evidence outside TikTok suggests that narratives can influence health knowledge and screening behavior [8, 9]. However, it is unknown whether narratives improve TikTok visibility or whether they are appropriate for every cancer topic. Recommendations involving patient narrators should therefore be tested prospectively and implemented with meaningful consent, privacy protections, and attention to whose experiences are represented. Public health agencies should similarly examine whether accurate cancer information remains competitive within an attention‐driven media environment rather than assuming accuracy and visibility naturally coincide. The higher share rate observed among medical creators may provide one useful outcome for such studies, but its meaning and downstream effects require direct evaluation.

Strengths of this study include the analysis of multiple cancer searches, separate examination of engagement components, reliable creator and content‐format coding, and sensitivity analyses using a more detailed creator classification. The four‐category analysis was particularly informative because it demonstrated how a coarse binary comparison can obscure meaningful heterogeneity within the reference group. Reporting both views per follower and adjusted absolute views also helped distinguish audience‐normalized accumulation from total observed exposure.

These findings should be interpreted within the limits of a cross‐sectional sample restricted to highly liked search results. This outcome‐conditioned sampling may limit generalizability and bias associations between creator characteristics and engagement. Ratio‐based measures, including views per follower, may reflect differences in their denominators as well as differences in reach or engagement and therefore are susceptible to ratio bias. We also could not distinguish follower from non‐follower views, measure unique reach, account for correlations among videos from the same creator, or observe algorithmic exposure. Professional roles were also self‐reported. Within these constraints, medical and nonmedical creators showed no clear difference in adjusted absolute views, but medical creators had lower views per follower and a higher share rate. Prospective studies with creator and viewer level data are needed to determine whether these patterns reflect differences in reach beyond established audiences and whether sharing ultimately expands the distribution of medical content.

## Author Contributions

**Eunice Y. Ju:** conceptualization, methodology, investigation, formal analysis, data curation, validation, visualization, writing the original draft, and reviewing and editing the manuscript. **Esther D. Ju:** methodology, investigation, formal analysis, validation, visualization, writing the original draft, and reviewing and editing the manuscript. **Russell G. Witt:** conceptualization, methodology, formal analysis, data curation, validation, visualization, resources, supervision, project administration, writing the original draft, and reviewing and editing the manuscript. All authors meet the ICMJE criteria for authorship, approved the final version of the manuscript for publication, and agree to be accountable for all aspects of the work.

## Funding

Dr. Russell Witt is supported by a National Cancer Institute Mentored Clinician Scientist Development Award under award number K08CA301024.

## Ethics Statement

This study analyzed publicly available data from TikTok content and did not involve interactions with users or collection of personal health information or identifiable private data. Public posts may nevertheless contain identifiable health disclosures. Usernames, URLs, screenshots, direct quotations, profile information, and other account identifiers were not collected and will not be shared in datasets. This study was approved by the University of Virginia Institutional Review Board Social and Behavioral Sciences.

## Consent

This study used only fully anonymized, publicly available data and involved no direct interaction with users. Informed consent was not obtained.

## Conflicts of Interest

The authors declare no conflicts of interest.

## Data Availability

The data that support the findings of this study are available from the corresponding author upon reasonable request.
